# Blood–brain barrier disruption in atrial fibrillation: a potential contributor to the increased risk of dementia and worsening of stroke outcomes?

**DOI:** 10.1098/rsob.200396

**Published:** 2021-04-21

**Authors:** Ritambhara Aryal, Adjanie Patabendige

**Affiliations:** ^1^ Brain Barriers Group, School of Biomedical Sciences and Pharmacy, The University of Newcastle, Callaghan, NSW 2308, Australia; ^2^ Brain and Mental Health Research Programme, Hunter Medical Research Institute, Newcastle, Australia; ^3^ Institute of Infection and Global Health, University of Liverpool, Liverpool, UK

**Keywords:** blood–brain barrier, atrial fibrillation, dementia, cognitive impairment, stroke, cerebral blood flow

## Abstract

Atrial fibrillation (AF) has become one of the most significant health problems worldwide, warranting urgent answers to currently pending questions on the effects of AF on brain function. Recent evidence has emerged to show an association between AF and an increased risk of developing dementia and worsening of stroke outcomes. A healthy brain is protected by the blood–brain barrier (BBB), which is formed by the endothelial cells that line cerebral capillaries. These endothelial cells are continuously exposed to shear stress (the frictional force generated by blood flow), which affects endothelial cell structure and function. Flow disturbances as experienced during AF can disrupt the BBB and leave the brain vulnerable to damage. Investigating the plausible mechanisms in detail, linking AF to cerebrovascular damage is difficult in humans, leading to paucity of available clinical data. Here, we discuss the available evidence for BBB disruption during AF due to altered cerebral blood flow, and how this may contribute to an increased risk of dementia and worsening of stroke outcomes.

## Graphical abstract

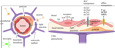

## 1. Introduction

Atrial fibrillation (AF) is the most common type of irregular heartbeat. Its prevalence is projected to double over the next two decades in people aged greater than 55 years [1]. AF is strongly associated with stroke, and there is an elevated risk of dementia in patients with AF who have a stroke [2,3]. Stroke is a leading cause of global death, affecting more than 13 million people annually [4]. Around one-third of all strokes is known to be caused by AF, and these strokes are more complicated than strokes which are not related to AF [5–8]. Studies have shown that AF increases the risk of stroke by three to fivefold [5,9–11]. In addition, around 19–30% of ischaemic stroke patients are reported to be affected by AF [12–14]. The interpretations of a 2015 meta-analysis indicate that the combination of various cardiac monitoring techniques has facilitated the detection of AF in almost one fourth of patients with stroke or transient ischaemia [13].

Importantly, both stroke and AF share the same risk factors such as age, diabetes, hypertension, heart failure, coronary heart disease and chronic kidney disease [7]. Cardioembolic stroke, which contributes greatly to mortality as well as permanent disability, is the most common subtype of stroke associated with AF [9]. Compared with 27% of non-AF-related strokes, there is a 50% probability that patients with stroke related to AF will demise within 1 year of the disease [9]. Patients with AF-related stroke have a 5-year survival rate of 39.2%, 5-year recurrence rate of 21.5% and 25.9% of these patients require nursing home care [15].

During AF, blood flow (and therefore shear stress) is altered, leading to the formation of blood clots, stroke, heart failure and death [16,17]. AF has been shown to be associated with cognitive impairment/dementia, independently of clinical cerebrovascular events (stroke or transient ischaemic attack). A recent study in over 6000 patients showed that AF contributed to the risk of dementia, beyond the risk attributable to anticoagulant use [18]. One of the plausible mechanisms is the occurrence of AF-induced changes in critical haemodynamic events causing brain vascular dysfunction.

Dementia is the term given to a group of conditions in which there is significant impairment in memory and one or more other cognitive domains that hamper an individual's capability to carry out daily activities. In a systematic review published in 2013, Kalantarian *et al.* [3] reported a significant association of AF with an increased risk of cognitive decline or dementia, either in the presence or in the absence of prior stroke. This meta-analysis proposed numerous mechanisms for the link between AF and cognitive decline. Some of these were common risk factors for AF and cognitive impairment such as diabetes, hypertension [19], hypercoagulation due to increased thrombin generation [20], periventricular white matter lesions [21] and silent stroke.

A recent study found higher levels of biomarkers of cerebral injury such as microtubule-associated Tau protein and glial fibrillary acidic protein (GFAP) in AF patients, suggesting potential blood–brain barrier (BBB, the protective physiological barrier of the brain) disruption, which could lead to cognitive decline or dementia in future [22]*.* Rusanen *et al.* [23] reported a link between AF and risk of dementia/Alzheimer's disease in late life but not in mid-life. By contrast, Bunch *et al.* assessed 37 025 patients from the Intermountain Heart Collaborative Study database, and reported that AF patients who were younger than 70 years old had the greatest risk of dementia. A 5-year follow-up assessment of this study found that patients with AF had several forms of dementia when compared with patients without AF [24]. Another study showed an increased risk of dementia in AF patients who were less than 67 years old [25]. Thacker *et al.* [26] observed a decline of cognitive scores at earlier ages in people with AF than those without AF. A Swedish study recorded dementia in 6.2% of AF patients from the year 2001 to 2007 (*n* = 12 057, age ≥ 45 years). In addition, this study also reported that female AF patients who had hypothyroidism and were taking levothyroxine (a medicine used to treat hypothyroidism) had a decreased risk of dementia compared with other female AF patients who did not have hypothyroidism and were not taking levothyroxine [27].

However, the underlying mechanisms that contribute to the development of dementia and stroke in AF patients remain poorly understood. Among the plausible mechanisms, the effect of altered cerebral blood flow (CBF) during AF on the BBB has been the least investigated. This is most likely due to the evident concerns relating to direct measurement of the cerebral vascular system leading to the paucity of clinical data, difficulty in studying the BBB in animals, and the complexity of replicating the cerebrovascular system in culture.

Deterioration in neuroprotective BBB function plays a major role in the pathogenesis of the disease, since the BBB dynamically responds to many events associated with flow disturbances (e.g. focal ischaemia), free radical release and cytokine generation. Any condition that affects the functional integrity of the BBB will cause secondary effects on CBF and vascular tone, exposing the brain to further damage [16–18]. Abnormal flow patterns or flow cessation can lead to changes in shear stress or pulsatility. This can deteriorate the brain endothelium and lead to barrier impairment [28,29]. Thus, a significant role is played by biomechanical forces generated by blood flow in the induction of many BBB properties and in modulating endothelial function.

In this review, we summarize and analyse current evidence on the association between the development of dementia and worsening of stroke outcomes in patients with AF, and the contribution of the BBB disruption due to altered CBF in AF as a major factor in developing these pathologies.

## 2. The structure and function of the blood–brain barrier

**Figure 1.**
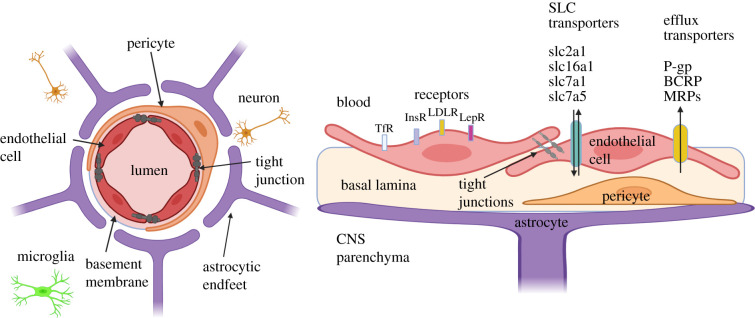
Healthy blood–brain barrier (BBB). The BBB consists of brain capillary endothelial cells joined by tight junctions, basement membrane, pericytes and astrocyte endfeet processes that surround the capillaries. These structures together with neurons and microglia form the neurovascular unit (NVU). The cross-section schematic on the right demonstrates various types of transporters and receptors expressed by brain endothelial cells. Active efflux transporters transport lipophilic molecules from the CNS towards blood: e.g. ABC transporters such as P-glycoprotein (P-gp), breast cancer resistance protein (BCRP) and multidrug resistance proteins (MRPs). The solute carriers (SLC) transport nutrients such as glucose and amino acids into the brain and can be unidirectional or bidirectional, e.g. GLUT1/SLC2A1 (glucose), EAAT1/SLC1A3 (glutamate), SLC16A1 (lactate, pyruvate), SLC7A1 (cationic amino acids) and LAT1/SLC7A5 (neutral amino acids). Several receptors are present in the BBB to meet the brain's metabolic demand: for example, transferrin receptor (TfR), insulin receptor (InsR), low-density lipoprotein receptor (LDLR) and leptin receptor (LepR). (Created with BioRender.com.)

The BBB is the structure, which separates the central nervous system (CNS) from the systemic circulation (figure 1). It is formed by brain capillary endothelial cells joined by complex tight junctions. The tight junction complex contains three main transmembrane proteins; namely occludin, claudins and junctional adhesion molecules (JAMs). The BBB is supported by a basement membrane, pericytes partially surrounding the endothelium and astrocyte endfeet processes that form a complex network around the capillaries [30,31]. Recently, the term ‘neurovascular unit’ (NVU) has been extensively used for the collective description of these structures along with neurons, microglia and other peripheral immune cells [32–34].

The basement membrane is a form of extracellular matrix, which surrounds the vascular tube. The brain contains two types of basement membrane: the inner vascular basement membrane, secreted by endothelial cells and pericytes, and the outer parenchymal basement membrane, secreted by the astrocyte endfeet processes. The basement membrane predominantly contains extracellular matrix proteins such as collagen IV, laminin, nidogen and perlecan [35,36].

Unlike highly permeable systemic capillaries, the tight junctions of the BBB seal the paracellular pathway and strictly control the passage of substances between the brain and the circulation at the brain capillary level. Therefore, almost all molecular traffic has to use the transcellular pathway, controlled by an array of transporters, receptors and enzymes (figure 1) [37]. Such an arrangement allows the entry of important molecules like water, gases, glucose and amino acids, while preventing the entry of pathogens, potential neurotoxins and xenobiotics and protects the brain from fluctuations in plasma composition [31,38,39].

The CNS endothelial cells express two major types of transporters: efflux transporters and solute carrier (SLC) transporters. Efflux transporters such as ABC (ATP-binding cassette) transporter family members transport lipophilic molecules from the CNS and the endothelium towards blood. They are predominantly expressed on the luminal membrane. These active efflux pumps protect the brain by removing endogenous neurotoxins and xenobiotics. However, many potentially neuroprotective drugs may also be substrates for these efflux transporters, leading to reduced brain penetration [40,41]. On the other hand, the SLC transporters enable the transport of various solutes and nutrients such as glucose and amino acids into the brain. SLC transporters are expressed on the luminal or abluminal membranes or both. Therefore, they are able to transport polar molecules, which cannot diffuse across the BBB, into and out of the brain, with the direction of the transport determined by the orientation of the transporter [36,37,42].

Contractile proteins possessed by pericytes enable the contraction of these cells to control the diameter of the capillary and regulate cerebral blood circulation [36,43,44]. Where the basement membrane is not present, pericytes and endothelium interlock to form peg-and-socket junctions. These junctions contain various transmembrane adhesion proteins, such as N-cadherin and connexin-43 [36,45,46]. Pericytes also play a crucial role in the activation of angiogenesis in the adult CNS, clearance of toxic substances, recruitment of immune cells into the brain and can act as multipotent stem cells to differentiate into various cell types of the CNS [36,44,47]. Hence pericytes are known to be important for the maintenance of the BBB during all stages (i.e. during the development of the BBB, adulthood and ageing [36,48]).

To meet the metabolic requirements of neurons, astrocytes facilitate the delivery of oxygen and glucose from the vasculature to neurons [49,50]. Astrocytes are also important for the maintenance of the BBB. By extending from the cell bodies to the basement membrane and towards the neurons, astrocyte endfeet enable bidirectional communication between the CNS vasculature and neurons [51,52]. Astrocyte endfeet secrete vasoactive substances such as prostaglandin E2 (PGE2) and epoxyeicosatrienoic acids (EETs), hence resulting in vasodilation and increased cerebral blood circulation [53–56]. Furthermore, astrocyte endfeet processes contribute to the BBB integrity by forming a structural border called glia limitans between the CNS neural tissue and non-neural cells. This barrier restricts the entry of leucocytes and other inflammatory cells from non-neural cells into the CNS parenchyma [57–59]. Astrocytes also respond to CNS injury via a process called astrogliosis or glial scarring, in which reactive astrocytes upregulate the expression of intermediate filament proteins such as GFAP, nestin and vimentin [60,61]. Furthermore, reactive astrocytes not only secrete cytokines such as interferon gamma (IFN-γ), tumour necrosis factor alpha (TNF-α) and interleukin-17 (IL-17), which modulate pro-inflammatory phenotypes of T-lymphocytes, but also anti-inflammatory cytokines such as interleukin-10 (IL-10) [62–64].

## 3. Mechanisms of blood–brain barrier disruption

The BBB disruption is evident in many neurological disorders, including dementia due to Alzheimer's disease [31,65,66], multiple sclerosis [67,68], Parkinson's disease [69,70], amyotrophic lateral sclerosis [71,72], Huntington's disease [73,74] and stroke [75–78]. In addition, emerging evidence suggest that peripheral diseases such as AF and others (e.g. heart failure, hypertension and diabetes mellitus) are also associated with BBB dysfunction [79–81].

**Figure 2.**
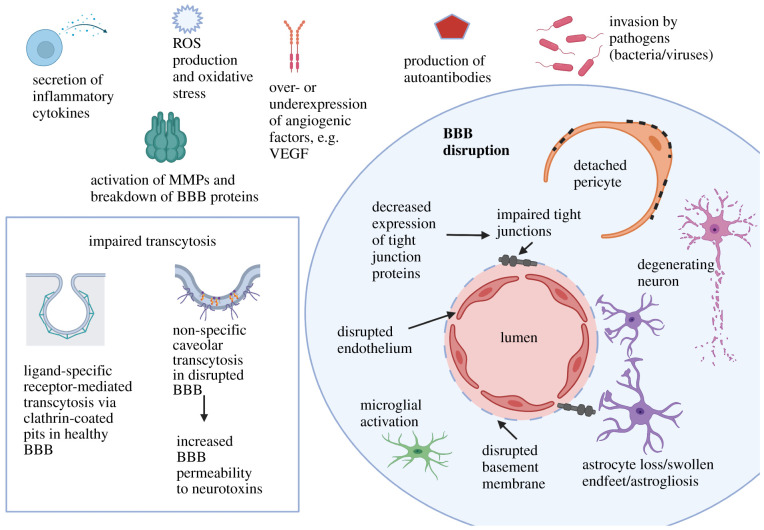
Blood–brain barrier (BBB) disruption. Several factors are associated with BBB disruption, some of which are illustrated here. Increased oxygen demand in the brain makes it susceptible to the production of reactive oxygen species (ROS), subsequent oxidative stress and activation of matrix metalloproteinases (MMPs). Likewise, over- or under-expression of angiogenic factors, production of autoantibodies, invasion by various pathogens and secretion of inflammatory cytokines are associated with impairment of BBB. Impaired transcytosis is another important factor that accompanies BBB disruption, in which the non-specific caveolar transcytosis takes over ligand-specific receptor-mediated transcytosis. Other characteristics of impaired BBB include impaired endothelium, detached pericytes, disrupted basement membrane and altered expression of tight junction proteins resulting in weakened tight junctions. Microglia, the inherent immune cells of the CNS increase their activity (microgliosis) and so do the astrocytes (astrogliosis). A compromised BBB loses its ability to restrict the entry of toxins and control brain homeostasis, and can lead to neurodegeneration, which is implicated in several neurological disorders. (Created with BioRender.com.)

As reviewed elsewhere, an intact BBB is essential for appropriate synaptic function, neuron connectivity and information processing [82–84]. Dysfunction of the BBB can lead to the entry of toxic substances and microbial agents into the brain and disrupt brain homeostasis. A damaged BBB can result in the activation of inflammatory and immune cells, leading to neuronal injury. Dysfunction of the BBB is associated with various factors such as oxidative damage due to reactive oxygen species (ROS) [85,86], secretion or activation of matrix metalloproteinases (MMPs) [87,88], angiogenic factors [89,90], autoantibodies [91,92] and pathogens [38,93] (figure 2).

Studies using different experimental animal models during the 1980s demonstrated that several compounds have the ability to affect BBB permeability. The complement activation protein 5a was shown to affect the integrity of endothelial cells and astrocytes [94]. Cerebral exposure to bradykinin results in the activation of the arachidonic acid cascade, which can result in altered BBB permeability [95,96]. Likewise, changes in Ca^2+^ influx across the BBB [97], the presence of serotonin receptors in the endothelial cells and signalling of histamine receptors H1R, H2R, H3R and H4R in neurons, astrocytes or endothelial cells can influence the BBB permeability [98,99]. Decreased levels of tight junction proteins such as occludin, claudin-5 and zonula occludens 1 (ZO-1) are linked with increased BBB permeability in Alzheimer's disease and hypoxia/ischaemia [100].

Impaired transcytosis (a process by which large molecules enter the CNS by crossing the BBB) is one of the features of BBB disruption [101]. Brain endothelial cells of healthy adults have an increased expression of receptors and components of clathrin-coated pits to facilitate the transfer of circulatory proteins through receptor-mediated transcytosis (RMT). Degeneration of pericytes with age promotes calcification of the vasculature and a shift from ligand-specific RMT to non-specific caveolar transcytosis (figure 2). This can potentially lead to neuroinflammation due to increased BBB permeability to neurotoxic proteins, including autoantibodies, albumin and fibrinogen [102,103]. An increase in the number of endothelial caveolae and the rate of transcytosis at the early stages of stroke, followed by disruption in paracellular barrier mechanisms have been shown to contribute to BBB dysfunction in stroke [104]. Furthermore, the expression of molecules such as Caveolin-1 and Plasmalemma vesicle-associated protein (PLVAP), which are important in vesicular transport during transcytosis, is upregulated in the impaired BBB [36].

The human apolipoprotein E (ApoE) facilitates the uptake of lipoproteins and promotes lipid metabolism. An absence of astrocyte-derived ApoE and the expression of ApoE4, a chief genetic risk factor for Alzheimer's disease leads to a compromised BBB [105,106]. Bell and colleagues reported that ApoE activates a pro-inflammatory Cyclophilin A-nuclear factor-kB-MMP-9 pathway in pericytes, which results in BBB disruption. This is followed by the uptake of neurotoxic proteins by neurons and a decrease in CBF [107]. Likewise, other studies have proposed loss of pericytes as a precondition for BBB disruption in some chronic conditions such as diabetes [108,109]. In human immunodeficiency virus (HIV) associated dementia, early inflammation of CNS, presence of perivascular macrophages and infiltration of monocytes are associated with BBB breakdown [110,111].

The presence of antioxidants such as glutathione, glutathione peroxidase, glutathione reductase, catalase and superoxide dismutase provide cerebral endothelium defence against oxidative damage [112,113]. However, the BBB integrity is compromised in the event of oxidative stress, as observed in several pathological conditions including various neurological disorders [86,114–116]. Increased oxygen demand for neuronal activity makes the brain susceptible to the production of ROS. High mitochondrial concentration in the endothelium also makes the brain vulnerable to increased oxidative stress. Likewise, the presence of nitric oxide (NO) in the brain can result in the generation of ROS [112]. Furthermore, the movement of the transmembrane protein occludin away from the tight junction has been shown to associate with oxidative stress-related BBB permeability [117]. Superoxide and its derivatives can cause vasodilatation by opening the potassium channels [118]. Other triggers of BBB disruption by ROS are activation of MMPs, autophagy and inflammation [119].

Shear stress or fluid shear stress is a tangential force of blood flow on the vascular endothelium. Several *in vitro* BBB models are available for studying the effects of shear stress on the BBB [120–123]. As reviewed by Wang *et al.* [121], shear stress under physiological conditions promotes BBB integrity by suppressing nicotinamide adenine dinucleotide phosphate hydrogen (NADPH) activation and ROS production, upregulating the expression and assembly of tight junction proteins, e.g. via VE-cadherin/Tiam1/Rac1 pathway. However, high shear stress during pathological conditions activates the release of MMPs such as MMP-2 and MMP-9 by platelets, which leads to the depletion of extracellular matrix and tight junctions. In addition, the activation of Src/ERK1/2 signalling pathway due to increased shear stress can lead to the downregulation of tight junction markers. Janigro and colleagues [28,124,125] have studied the effects of shear stress on the BBB extensively using human brain endothelial cells co-cultured with human astrocytes in a dynamic *in vitro* BBB (DIV-BBB) model. They have shown that shear stress leads to tightening of the BBB, increase in the expression of multidrug resistance transporters, ion channels, several cytochrome P450 enzymes and SLC transporters, leading to differentiation of endothelial cells into a BBB phenotype while inhibiting cell proliferation [28].

Elbakary & Badhan [126] used the Quasi-Vivo 600 (QV600) interconnected chamber system to perfuse media and apply shear stress on high transendothelial electrical resistance (TEER) primary porcine brain microvascular endothelial cell (PBMEC) cultures [127]. The application of shear stress on these cells caused reorientation and improvement of tight junction formation along with an increase in TEER, in comparison with the cells which were cultured under static conditions. In another study, shear stress was applied on confluent monolayers of derived human brain microvascular endothelial cells (dhBMECs) [128]. These cells displayed a distinctive phenotype compared with cells grown under static conditions, such as the absence of elongation and alignment, significant reduction in rates of apoptosis and proliferation, and unaltered expression of important BBB markers. Garcia-Polite *et al.* [29] observed that the expression of tight junction markers such as ZO-1 and claudin-5 was upregulated by more than 1.7 fold by physiological shear stress, whereas their expression was reduced to basal levels by increased shear stress.

One important consideration regarding the factors/events associated with BBB disruption is that they can either precede or succeed BBB damage or both. In other words, some factors fit into the categories of both ‘causes’ and ‘consequences’ of BBB disruption. For instance, entry of pathogens causes BBB disruption leading to increased BBB permeability [129], impaired tight junctions [130] and disrupted basement membrane [93], which can facilitate further entry of other pathogens and toxic substances (consequence). Likewise, the release of inflammatory cytokines in response to CNS injury (cause) leads to BBB impairment, which can result in microglial activation [131,132]. Activated microglia may in turn release more cytokines/chemokines (consequence) and lead to neuronal damage. In addition, activated microglia have the ability to produce ROS [133], the latter of which is also a causal factor for BBB impairment. This could also be true for other factors listed above, which are known to be contributary to BBB leakage. For example, peripheral or CNS cytokines may induce BBB leakage in neuropsychiatric lupus, hence allowing the entry of autoantibodies via disrupted BBB into the CNS [134] (see review by Obermeier *et al.* [38] for a discussion on how BBB disruption can cause or contribute to neurological disease).

In addition to the above mechanisms, there is a plethora of literature on other means of BBB disruption. Herein, we will focus specifically on how CBF changes are associated with BBB damage.

## 4. Effects of altered cerebral blood flow on the blood–brain barrier

To match its metabolic requirements, the mammalian brain contains an exceptional mechanism of regional CBF regulation and neuronal activation termed ‘neurovascular coupling’ (NVC). Interaction of various cells and components of the BBB contributes to this process [135–137]. NVC, also known as functional hyperemia, starts with alterations in neuronal activation, which modulates vasodilation and eventually leads to an increase in CBF. Studies have shown that NVC occurs even if there is a reduction in neuronal activity, and it is facilitated by the action of excitatory as well as inhibitory neurons [135,138,139].

The brain also has a regulatory mechanism called cerebral autoregulation, which ensures appropriate CBF and oxygen transport over differing perfusion pressures [140,141]. Normal CBF is maintained at around 50 ml per 100 g of brain tissue per minute, within the range of 50–150 mmHg of cerebral perfusion pressure (the difference between intracranial pressure and mean arterial pressure) [142–144]. When this pressure falls below 50 mmHg, the CBF decreases, leading to ischaemia, whereas rise of pressure above 150 mmHg results in cerebral oedema and BBB breakdown [144]. Cerebral autoregulation circumvents perfusion abnormalities and reduces the subsequent risk of haemorrhage or ischaemia.

Two types of cerebral autoregulation have been described: blood flow alterations during rapid changes in blood pressure are called dynamic cerebral autoregulation, and blood flow changes in response to continued blood pressure changes are called static cerebral autoregulation [145,146]. An absence of autoregulatory mechanisms can lead to significant brain injuries. For instance, forced opening of cerebral vessels during acute hypertension results in a massive increase in CBF (300–400%) in a phenomenon called autoregulatory breakthrough. Reduced cerebrovascular resistance also raises hydrostatic pressure on endothelial cells, resulting in the development of oedema [142,147,148]. Castro *et al.* [144] outlined that cerebral autoregulation is disrupted focally in large vessel stroke and globally in small vessel stroke. Moreover, transcranial Doppler (TCD) studies have shown gradual degradation in cerebral autoregulation after 5 days of stroke with a recovery period of around three months [145,149,150]. While a normal CBF pattern safeguards proper functioning of the brain, abnormalities in CBF resulting from the disruption of cerebral autoregulation and neurovascular coupling is linked with BBB disruption in disease conditions such as ischaemic stroke, hypertension, Alzheimer's disease and AF [140,151].

The CBF is regulated by factors such as partial pressure of CO_2_ and O_2_, cerebral metabolism and the autonomic nervous system. Other substances such as glutamate activate neuronal *N*-methyl-d-aspartate (NMDA) receptors, which in the presence of ample glucose and oxygen further activates NO synthase that produces NO, a vasodilator of parenchymal arterioles [135,152]. Astrocytes respond to glutamate release from synaptic terminals by producing the vasoconstrictor, arachidonic acid and parenchymal arteriole dilators such as EET and prostaglandins [153,154]. Moreover, the dilatation of cerebral arterioles caused by the activation of ATP-sensitive K^+^ channels during low oxygen conditions (hypoxia) is associated with an increase in CBF [142,155]. In a study using mice pups, the transitory opening of BBB was reported in response to hypoxia–ischaemia [156]. Likewise, the lowering of CO_2_ level in the blood (hypocapnia) has been known to promote vasoconstriction and a subsequent reduction of CBF. On the other hand, elevated CO_2_ level in the blood (hypercapnia) causes vasodilation and successive augmentation of CBF [142,157]. A recent study described that hypercapnia worsens BBB disruption by promoting the overproduction of interleukin-1β in blood [158].

Direct assessment of the effects of changes in CBF on brain function is challenging. However, various techniques are available for the assessment of CBF such as arterial spin labelling/pulsed arterial spin tagging and vascular-space occupancy [159]. Shen & Duong [160] have reviewed some technical developments of CBF measurement methods in animal models. One technique is inversion-recovery background suppression continuous arterial spin labelling (IR-cASL), which shows 2 times improved temporal stability and 2–2.3 times higher functional contrast-to-noise ratios for stimulation of hypercapnia in rats, in comparison with cASL without background suppression. Furthermore, this technique was used to demonstrate that whole-brain CBF values in these animals were similar across various labelling periods [161].

High spatial resolution CBF magnetic resonance imaging (MRI) is another technique that can provide valuable information on brain CBF supply at columnar and laminar levels. Using this method, Shen *et al.* showed that the whole-brain CBF in rat brains was around 0.89 ml g^−1^ min^−1^. The maximum CBF values were found along the neocortex (1 ml g^−1^ min^−1^, within the range of 0.89–1.16 ml g^−1^ min^−1^) and the minimum CBF values were observed in the corpus callosum (0.32 ml g^−1^ min^−1^). These observations showed a CBF ratio of 3.1 for grey matter: white matter [162]. This method was also used to study rats with stroke before and after reperfusion. It was found that brain regions of normal perfusion and hypoperfusion were diverse during middle cerebral artery occlusion (MCAO) [160].

The cardiac spin labelling (CSL) technique allows higher sensitivity CBF imaging and measurement of cerebral, retinal and choroidal blood flow in small animals such as mice [160]. A two-compartment exchange model for quantification of perfusion, described by Zhou *et al.* [163] allows controlled trafficking of blood/water between the microvascular compartment (containing arterioles, venules and capillaries) and the extravascular space in the parenchyma. During limited water exchange, the FAIR (flow-sensitive alternating inversion recovery) signal intensities of two compartments could be compared in magnitude but they did not overlap in time. Whereas during fast limited water exchange, flows quantified from the signal-intensity difference were underestimated. This observation was found to be more significant for bigger flows and increased magnetic field strengths. Likewise, the near-infrared spectroscopy (NIRS) indocyanine green dye dilution technique allows non-invasive bedside measurement of regional CBF and regional cerebral blood volume [164]. Preliminary results showed that the values obtained from this technique matched with the values obtained from a standard method such as perfusion-weighted MRI.

## 5. Disrupted peripheral and cerebral blood flow in atrial fibrillation

As reviewed by Calenda *et al.* [165], histopathological features like an aggregation of glycogen granules in atrial cardiomyocytes, increased loss of sarcomeres and variable degrees of interstitial fibrosis are known to manifest as AF in the heart, along with some additional indicators [165,166]. These changes may have been contributed by various factors such as ageing, genetic predisposition, endothelial disruption, inflammation and stretching of the atrial wall [167,168].

Based on past and recent experimental studies, it has been widely acknowledged that AF can cause impairments in the coronary blood flow pattern in humans [169–171]. One study by Saito *et al.* [172] showed a significant reduction in coronary blood flow in dogs with artificially induced AF. Likewise, Kochiadakis *et al.* [173] reported an increased coronary circulation in patients with acute AF. While the mechanism of perturbed coronary circulation in AF remains debated, Scarsoglio *et al.* [174] proposed that impaired coronary blood flow was attributed to the higher ventricular rate during AF. Similarly, another study using coronary angiography showed that a fine fibrillary wave was a key factor for decreased coronary flow in AF patients, as compared to the sinus rhythm [175]. Apart from these studies on peripheral circulation, AF is also closely linked to blunted CBF as discussed below.

A recent study by Junejo *et al.* [140] reported reduced cerebral autoregulation and impaired neurovascular coupling response to visual stimulation in people with AF. This observation corroborates very early studies that date back to the 1980s. Lavy *et al.* [16] found a reduction in cerebral circulation in patients with AF. The rate of reduction in CBF was higher in patients of age group 35–50 years [17.5%] compared with the age group of 51–65 years (13.4%) and above 65 years (5.5%)*.* By contrast, a recent study by Gardarsdottir *et al.* [17] reported that the elderly population with persistent AF had reduced total CBF and brain perfusion*.* The authors speculate that this may be an explanation to their previous finding in which AF was associated with decreased brain volumes and cognitive decline in elderly patients with AF [176].

Measurement of CBF with the intravenous xenon-133 technique by Petersen and colleagues showed a decrease in CBF in AF patients who did not have heart failure. The CBF was increased in these patients subsequent to electrical cardioversion (a technique that uses electric current to readjust heart rhythm to normal sinus rhythm). This observation led the authors to speculate that a decrease in CBF can cause recurrent cerebral abnormalities associated with AF [177]. Another study used TCD sonography to measure CBF in patients with paroxysmal AF. They observed that the mean flow velocity significantly decreased in the middle cerebral artery but not in other arteries [178]. Neuropsychological testing and TCD procedure in patients with heart failure indicated cerebral hypoperfusion as a plausible mechanism that contributes to the worsened cognitive function in these patients [179]. Likewise, recent advances in computational algorithms have reinforced the hypothesis of altered CBF dynamics in AF, marked by hypertensive events and transitory hypoperfusions [180].

To better understand the haemodynamics during AF, some researchers have developed computational models that imitate AF. Choi and colleagues performed computational fluid dynamics (CFD) simulations to comprehend the course of blood clots in the aortic arch under normal and AF conditions. This study showed that the frequency of blood clots is significantly increased in AF when compared with the aortic flow at normal conditions. The authors suggest that this type of computational approach would be valuable in determining the effect of AF cardiac haemodynamics in the cerebral embolization of blood clots, which could result in stroke [181]. Another computational study used two coupled lumped-parameter models of cardiovascular and cerebrovascular circulations to mimic AF and sinus rhythm. There were increased cerebral haemodynamic variations (such as brief periods of decreased blood flow or increased pressure) in the distal circulation under the conditions of AF, which was proposed to be a mechanism that initiates cognitive impairment or dementia in AF patients [182]. A similar approach was used by Scarsoglio *et al.* [183,184] where they noted differences in artificially built signals gained from their computational model in the direction of distal cerebral regions in AF compared with normal sinus rhythm.

Oral anticoagulants (e.g. warfarin), which are used for stroke prevention in AF patients, have also been described to be effective in dementia prevention, although this remains contentious [18,185–187]. The CHADS2 and CHA_2_DS_2_VASc scores not only predict the risk of stroke in AF patients, but they also indicate the risk of dementia in these patients [18,188,189]. Graves *et al.* [18] reported that in patients with long-term warfarin treatment, the presence of AF showed an increased risk of dementia across all CHADS2 score levels compared with patients without AF. The authors speculated that dementia risk contributed by AF is not exclusively caused using anticoagulation therapy, and AF may facilitate the development of dementia towards the end-stage of the disease [18]. Friberg & Rosenqvist [190] reported that early anticoagulation treatment decreased the risk of dementia by 29% in patients with AF compared with patients without anticoagulation treatment. In another study, Jacobs *et al.* [191] discovered that both over-coagulation and under-coagulation treatments increased the risk of dementia, hence signifying chronic cerebral injury as a factor that links AF and dementia.

Animal models of AF can only reproduce certain but not the entire spectrum of human AF. AF induction in these animals lasts for a very short time. Animals that have been used to study AF include mouse, rat, guinea pig, rabbit, goat, dog, sheep, pig and horse [192]. Corday and colleagues measured CBF in dogs using a magnetic probe which was connected to a square wave electromagnetic flowmeter and integrator-computer. A mean reduction was observed in CBF by seven per cent in premature atrial systoles, 12% in premature ventricular systoles and 23% during AF. The authors also highlighted that cerebral angiospasm can occur during the arrythmias, which may continue afterwards [193]. Likewise, Friedman *et al.* [194] observed reduced CBF in the cerebellum and brain stem, along with a decrease in splanchnic and renal cortical blood flow in dogs with AF. These studies implicate that cardiac arrhythmias may cause an ample decrease in CBF which can ultimately lead to cerebral ischaemia [193].

To our knowledge, there have been very limited experimental studies on animal models of AF, other than those mentioned above, which have focused on the mechanisms of BBB disruption or impaired cerebral circulation. Future studies are urgently required to get a better understanding of the underlying mechanisms that can help develop new treatments.

## 6. What are the underlying mechanisms that increase the risk of stroke and dementia in atrial fibrillation?

In this review, we provide a summary of our current understanding of AF, stroke and dementia associations, and demonstrate how changes in CBF due to AF could disrupt the BBB, leading to worsening or developing stroke and dementia. Old age is a major shared risk factor for dementia, stroke and AF, although these conditions are also observed in the young age group [185,195]. While AF significantly increases the risk of stroke, it is also extremely important to highlight the fact that stroke patients have a higher risk of cognitive impairment/dementia. Development from AF to cognitive decline or dementia can occur either directly or with stroke as an intermediate [196]. Furthermore, several studies have proposed BBB disruption as a harbinger of cognitive dysfunction [197–199].

Hence either the coexistence or co-influence of these three diseases is highly likely. Very little is known about the potential mechanisms via which AF contributes to cognitive impairment/dementia and clinical stroke. We propose that changes in CBF patterns and BBB disruption during persistent AF may lead to increased risk of dementia and worsened stroke outcomes.

Our hypothesis is based on the observations of studies that showed direct or indirect impairment of BBB/CBF during AF. One such study is the work of Junejo *et al.* [140], which indicated that cognitive dysfunction might result from a weakened cerebrovascular regulation. Bunch *et al.* [200] also proposed that cerebral microvascular dysfunction may be responsible for sudden cognitive decline in patients with AF. This concept is bolstered by the observation of Bangen *et al.* that cerebral atherosclerosis was the chief cerebrovascular pathology in the brain autopsies of 84 Alzheimer's disease patients. Atherosclerosis was predominantly detected in the circle of Willis, a region where several arteries join at the inferior side of the brain, to allow collateral blood flow between anterior and posterior aspects of the brain. The authors proposed that maintaining proper cerebrovascular health could result in averting or delaying dementia in people with Alzheimer's disease pathology [201].

Two other studies noted increased plasma levels of von Willebrand factor, a biomarker of endothelial dysfunction in patients with AF, which could predict stroke and cardiovascular risk [202,203]. Freestone *et al.* [203] also reported that patients with chronic AF exhibit impaired brachial artery flow-mediated dilatation (FMD) response. This is suggestive of diminished endothelial NO bioavailability [140,204,205] and possible perturbation of CBF regulation [206]. Besides markers of endothelial dysfunction, biomarkers of oxidative stress and inflammation are known to be raised in AF as well as in Alzheimer's disease, which are also evident in BBB disruption [200,207].

Some studies have shown that the treatments for abnormal heart rhythms can also be effective in improving cognition. Efimova *et al.* [208] investigated the effects of atrioventricular node ablation and pacemaker implantation in brain perfusion and cognitive function of patients with AF. Not only did the authors note improved ventricular systolic function, but also enhanced blood perfusion in temporal and frontal cortices, improved verbal and visual memory and advanced learning. Likewise, Bunch *et al.* [209] reported that AF patients who were treated with catheter ablation had lesser rates of dementia and stroke than the patients who were not treated with catheter ablation for AF.

A systematic review and meta-analysis of studies from 1950 to 2009 described that ten per cent of the population suffered from dementia before they had their first stroke. Furthermore, ten per cent of patients suffered from new dementia shortly after their first stroke attack, and over one-third of the population suffered from dementia succeeding recurrent stroke. In addition, pre-stroke dementia was linked with risk factors such as the family history of dementia, female gender and medial temporal lobe atrophy, whereas post-stroke dementia was associated with multiple cerebral lesions. Hence, prevention of stroke or proper care after episodes of stroke seems to be crucial in reducing the risk of dementia [210].

While many studies have supported the association between AF and dementia risk in patients with stroke [2,211,212], a meta-analysis by Kwok *et al.* [2] pointed out the ambiguity on such an association in wider population. Reports have also shown that patients with AF have an increased risk of cognitive deterioration/dementia even in the absence of medical history of previous stroke [213–215]. There is increasing evidence that silent cerebral infarcts (SCI) are commonly observed in AF patients [5,216,217]. A meta-analysis reported that MRI detected SCI in 40% of patients with AF without a history of symptomatic stroke [216]. Likewise, Hahne *et al.* [5] reviewed that the rates of SCI were 12.3–92% in AF patients and 17–69% in non-AF patients, and the rates of silent strokes increase following AF ablation procedures.

Petersen *et al.* [217] observed that SCIs were mainly present in the cortex , and the AF group had a higher number of SCIs than the control group; however, there was no significant difference in the size of SCIs between the two groups. Studies show that the risks of symptomatic stroke and dementia increase by more than threefold [218] and twofold [212], respectively, in the presence of SCI. In addition, other studies have also considered SCI as a risk factor for future cognitive dysfunction and dementia [212,219] as well as subsequent stroke [220]. Hence Kalantarian *et al.* [216] proposed that a high prevalence of SCI in AF patients may make these patients vulnerable to cognitive decline/dementia and future stroke. This finding is in line with our notion that altered CBF due to cerebral infarction could be one factor which links AF, stroke and dementia together.

Manifestation of other Alzheimer's disease-related pathologies in stroke or AF have also been demonstrated. For example, ApoE4, the major genetic risk factor of Alzheimer's disease has been recently shown to be involved in the escalation of dementia prior to or following transient ischaemic attack and stroke [221,222]. Cerebral atrophy or decreased brain volume is another major characteristic of Alzheimer's disease. Reduced brain volumes are not only observed in stroke [223,224] but also in AF patients, especially in persistent AF compared with paroxysmal AF patients [176,225]. As vascular impairment is associated with cerebral atrophy (e.g. in vascular dementia), this reinforces our hypothesis that cerebrovascular/BBB dysfunction could be one of the regulators of cerebral atrophy in dementia, stroke and AF.

Furthermore, myelin degeneration or demyelination is another common observation in Alzheimer's disease, vascular dementia [226–228] and stroke [229–232]. Case studies have previously reported that multiple sclerosis, a demyelinating disease is associated with AF and electrocardiographic changes in some patients [233,234]. These studies suggested that demyelination could alter cardiac conductance causing arrhythmias and result in neurogenic pulmonary oedema. In fact, demyelination is strongly linked with BBB impairment in multiple sclerosis and some other CNS diseases [235–238].

## 7. Concluding remarks

**Figure 3.**
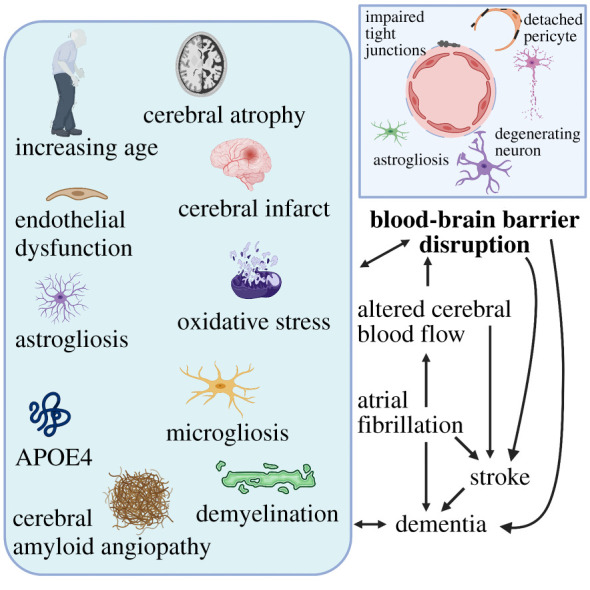
Blood–brain barrier (BBB) disruption in atrial fibrillation. The risk of atrial fibrillation (AF), stroke and cognitive impairment/dementia increases with age. Altered blood flow due to AF increases the risk of stroke, and stroke increases the risk of dementia. The progression of AF to dementia can be either direct or via transitional stroke. One of the plausible mechanisms is the occurrence of AF-induced changes in critical haemodynamic events causing BBB dysfunction. The box on the left lists some events associated with BBB disruption, all of which are evident in AF, stroke and dementia. Development of therapeutics that protect the BBB may help prevent or delay AF-related neurological complications more effectively. (Created with BioRender.com.)

This review highlights a critical gap in the current knowledge in understanding neurological manifestations in AF, and the need to address the missing link—a disrupted BBB (see figure 3). Protection of which can lead to improved outcomes for AF patients and stop or delay the progression of developing dementia and stroke. However, there are important unanswered questions. There is an urgent need for further studies and the development of suitable models for assessing BBB disruption to determine the underlying mechanisms. These are outlined in box 1. While several BBB models are available for assessing BBB disruption [121,239], a lack of suitable models for investigating how different environmental stimuli such as changes in CBF or blood-borne substances can affect the BBB during AF, leading to dementia or stroke, is a major hurdle. Developing therapeutics to protect the BBB needs to be a priority to help prevent AF-related neurological complications more effectively.

Box 1. **Outlook**

1. Need for further studies:

— More studies on improved risk stratification scores and screening tools are needed for the detection of dementia/stroke in AF patients [240].

— Need ample evidence from larger cohorts regarding the effects of oral anticoagulation/catheter ablation on cognitive function in AF/stroke; likewise, more studies are needed to determine the effects of lifestyle changes/dietary interventions on cognitive function in AF/stroke [241].

— Efficacy of anticoagulation on SCI is not clear yet as shown by some studies [242,243]. Future studies need to check whether anticoagulation results in reduced incidence of SCI, and if this in turn leads to decreased incidence of dementia, symptomatic stroke and mortality [216].

— More studies are needed to determine the best biomarkers for predicting stroke and response to anticoagulants [244].

2. Challenges:

— There is a paucity of information on the association of altered CBF dynamics and AF. As put forward by Saglietto *et al.* [180], this is attributed to issues regarding direct sampling of the cerebral circulation and the lack of resolving power to get information on pressure and flow signals downstream the cerebral arteries.

— Dementia may cause reduced adherence to oral anticoagulation therapy in AF/stroke [186].

— Improved strategies for AF detection needed as AF is undetected and untreated in many patients since they do not show symptoms, or they have paroxysmal AF [245].

3. Availability and suitability of current models and methods for assessing BBB dysfunction due to changes in CBF:

— A range of *in vitro* BBB models has been developed over the years [246,247]. These include *in silico* models, microfluidic systems, ECM-based models, Transwell systems, spheroidal models and isolated brain microvessels. However, not all models are suitable for elucidating the mechanisms of flow-induced changes.

— There is no ‘gold-standard’ BBB system that perfectly imitates the human BBB. For example, brain endothelial cell monolayers, non-contact Transwell cocultures and monoculture microfluidic models have limited cell interactions; the BBB-on-a-chip model has limited reproducibility of cell phenotype [248]. Dynamic flow-based models or microfluidic models that incorporate brain endothelial cells as well as astrocytes, pericytes and neurons are more suitable for investigating the role of BBB during disrupted CBF in AF [121]. However, these models are more expensive and can be labour-intensive.

— MRI-based methods to assess BBB function in AF or computerized simulation of CBF to determine BBB disruption can be other options that need further validation.

4. Potential treatments focusing on protecting BBB:

— BBB dysfunction hinders the transport of amyloid plaques, the major pathological hallmark of Alzheimer's disease, from the brain to the peripheral circulation. Hence potential treatments focusing on BBB protection could be beneficial in the treatment of Alzheimer's disease [65].

— Manipulation of BBB could be used for targeted drug delivery for the treatment of neurodegenerative disorders [31], e.g. reversible BBB disruption by chemical or physical methods to allow entry of drugs; drug lipidization for increased permeability, development of more hydrophobic drug analogues [249].

## Data accessibility

This article has no additional data.
